# Effect of Acid Hydrolysis Conditions on the Properties of Cellulose Nanoparticle-Reinforced Polymethylmethacrylate Composites

**DOI:** 10.3390/ma7010016

**Published:** 2013-12-20

**Authors:** Guangping Han, Siqi Huan, Jingquan Han, Zhen Zhang, Qinglin Wu

**Affiliations:** 1 Key Laboratory of Bio-based Material Science and Technology (Ministry of Education), Northeast Forestry University, Harbin 150040, China; E-Mail: huansiqi888@hotmail.com; 2 School of Renewable Natural Resources, Louisiana State University Agricultural Center, Baton Rouge, LA 70803, USA; E-Mails: hjingq1@tigers.lsu.edu (J.H.); zzhan39@lsu.edu (Z.Z.); wuqing@lsu.edu (Q.W.)

**Keywords:** cellulose nanoparticles, PMMA, thermal expansion, mechanical properties

## Abstract

Cellulose nanoparticles (CNPs) were prepared from microcrystalline cellulose using two concentration levels of sulfuric acid (*i.e.*, 48 wt% and 64 wt% with produced CNPs designated as CNPs-48 and CNPs-64, respectively) followed by high-pressure homogenization. CNP-reinforced polymethylmethacrylate (PMMA) composite films at various CNP loadings were made using solvent exchange and solution casting methods. The ultraviolet-visible (UV-vis) transmittance spectra between 400 and 800 nm showed that CNPs-64/PMMA composites had a significantly higher optical transmittance than that of CNPs-48/PMMA. Their transmittance decreased with increased CNP loadings. The addition of CNPs to the PMMA matrix reduced composite’s coefficient of thermal expansion (CTE), and CNPs-64/PMMA had a lower CTE than CNPs-48/PMMA at the same CNP level. Reinforcement effect was achieved with the addition of CNPs to the PMMA matrix, especially at higher temperature levels. CNPs-64/PMMA exhibited a higher storage modulus compared with CNPs-48/PMMA material. All CNP-reinforced composites showed higher Young’s modulus and tensile strengths than pure PMMA. The effect increased with increased CNP loadings in the PMMA matrix for both CNPs-64/PMMA and CNPs-48/PMMA composites. CNPs affected the Young’s modulus more than they affected the tensile strength.

## 1. Introduction

Cellulose is the most abundant renewable organic material produced in the biosphere, with an annual production estimated to be over 7.5 × 10^10^ tons [1]. Cellulose is widely distributed in plants and to a lesser degree in algae, bacteria, invertebrates, and protozoa [2]. It is well known that acid hydrolysis of cellulose fibers yields highly ordered rod-like cellulose nanocrystals (CNCs), also called nanocrystalline cellulose [3]. CNCs are highly crystalline with a width of 2 to 20 nm and a length up to several micrometers [4]. CNCs have high mechanical properties along the longitudinal direction with an estimated modulus of elasticity of 138 GPa [5]. The coefficient of thermal expansion (CTE) of CNCs along the longitudinal direction is less than 1 × 10^−7^ °C^−1^, which is as small as that of quartz [6]. These excellent features make cellulose microfibrils and nanofibers promising materials as the reinforcement in nanocomposites.

In the past few years, research on cellulose nanofibers (CNFs) has primarily focused on extracting CNFs from different plants [7–9] and using CNFs to reinforce various polymer matrices [10–12]. Yano *et al.* [13] reported a novel nanocomposite that could be used as the substrate for flexible Organic Light Emitting Diode (OLED). In their study, bacteria cellulose (BC) pellicles were immersed into neat acrylic and epoxy resin to make the nanocomposites. All the composites showed outstanding properties due to the strong reinforcement effect from BC. It has also been reported that by utilizing microfibrillated cellulose in the form of sheets impregnated with phenolic resin and compression under high pressure, it was possible to produce high-strength composites exploiting the unusually high strength of cellulose microfibrils. The mechanical properties of the novel composites were impressive, with a bending strength of as high as 370 MPa [14].

Many transparent polymeric materials, such as polymethylmethacrylate (PMMA), poly-carbonate, and polystyrene, have been widely used as optical materials because of their excellent optical clarity and low density. However, their application is restricted by their relatively low mechanical properties. Many attempts have been made to enhance their mechanical performance. Studies have focused on the formation of strong and transparent polymer composites using BC [15–17]. BC nanofibers have been acetylated to enhance the properties of optically transparent composites of acrylic resin reinforced with the nanofibers [18]. Reinforcements with nanoparticles [19], micro-sized fibers [20], and CNCs [21,22] have also been shown to be effective approaches for enhancing the mechanical properties of the transparent polymeric materials. Liu *et al.* [21] reported the improved mechanical properties of PMMA films by the addition of CNCs. The mechanical properties of the CNC/PMMA composites were reported to be largely related to the nature of the CNCs (dimension, shape, and aspect ratio) and the compatibility or interaction between these two polymers. Therefore, it is worthwhile to investigate the properties of the transparent plastics reinforced with different types of cellulose nanoparticles (CNPs).

The objectives of this study were to examine the optical, thermal expansion, and mechanical properties of PMMA-based nanocomposites reinforced with two kinds of CNPs (*i.e.*, CNCs and CNFs). The CNP-reinforced composite films were manufactured and the effect of CNP loading level on the performance of the composites was discussed.

## 2. Experimental

### 2.1. Raw Materials

Microcrystalline cellulose (MCC) (Nippon Paper Chemicals Co., Ltd., Tokyo, Japan) was used as the raw material for producing CNPs. Sulfuric acid (95 to 98 wt%, VMR, West Chester, PA, USA), N,N-dimethylformamide (DMF) (Sigma-Aldrich, St. Louis, MO, USA), and poly(methyl methacrylate) (PMMA) (*M*_w_ = 75,000, Polysciences Inc., Warrington, PA, USA) were of analytical grade and used as received without purification.

### 2.2. Preparation of CNPs

CNPs were prepared by sulfuric acid hydrolysis followed by high-pressure homogenization (HPH). Two types of CNPs were prepared using two different concentrations of sulfuric acid aqueous solution, (*i.e.*, 48 and 64 wt%). Fifteen grams of MCC were mixed with 400 mL of sulfuric acid aqueous solution, and the mixture was stirred vigorously at 45 °C for 1 h. Ten-fold dilution was then applied to the mixture to stop the hydrolysis reaction. The suspension was centrifuged at 12,000 rpm for 20 min (Sorvall ST 16R, Thermo Fisher Scientific Inc., Pittsburgh, PA, USA) to separate the CNPs in the suspension. The CNPs were then washed with distilled water; and the mixture was centrifuged and the CNPs separated again. This process was repeated 3 times. The precipitate was placed in regenerated cellulose dialysis tubes (Thermo Fisher Scientific Inc., Pittsburgh, PA, USA) with a molecular weight cutoff of 12,000 to 14,000 and dialyzed against distilled water for several days until the water pH reached a value of 7.0 [21].

To further reduce the size of the CNPs, the suspension of CNPs was processed through a high-pressure homogenizer (Microfluidizer M-110P, Microfluidics Corp., Newton, MA, USA) with a pair of Z-shaped interaction chambers (one 200 μm ceramics, and one 87 μm diamond) under an operating pressure of 207 MPa. The suspension was finally collected after five passes through the homogenizer. The obtained materials through 48 wt% and 64 wt% sulfuric acid hydrolysis and HPH were designated as CNPs-48 and CNPs-64, respectively. The CNPs were concentrated to 1.5 to 2.0 wt% dispersion in water and stored in a cold room until needed.

### 2.3. Dispersion of CNPs in DMF by Solvent Exchange

The CNPs-48 and CNPs-64 dispersed in H_2_O were solvent-exchanged into DMF solvent by vacuum-assisted rotary evaporation. A 200-mL aqueous dispersion of CNPs was placed into a flask, and an equal volume of DMF was added slowly under agitation. The H_2_O/DMF/CNP solution was then poured into a round-bottomed flask. Water and a small portion of DMF in the mixture were subsequently evaporated using a rotary evaporator until the amount of distillate was around 200 mL. To test whether there was residual water in the mixture, a small amount of the solution was pipetted out and mixed with PMMA. If the PMMA dissolved in the solution, the solvent exchange was completed. The weight percentage of CNPs in DMF was determined based on the dry weight of CNPs after evaporating the solvent at 60 °C to a constant weight.

### 2.4. Preparation of CNP/PMMA Composite Films

PMMA was dissolved in DMF solution to prepare a mixture with a concentration of 150 mg/mL. The required amount of CNPs in the DMF dispersion of known concentration was added to the PMMA/DMF mixture to obtain the desired CNP weight percentage in the subsequent CNP/PMMA films. The resulting mixture was intensely stirred to achieve a uniform dispersion of CNPs in the polymer matrix. Finally, the solution was cast in glass petri dishes and then dried in an oven at 50 °C for several days. Using CNPs at loadings of 0, 5, 10, 15, and 20 wt%, a series of composite films with a thickness of about 0.5 mm were prepared.

### 2.5. Transmission Electron Microscopy (TEM)

For the TEM analysis, concentrations of the aqueous CNP suspensions were diluted to 0.05 to 0.1 wt%. The diluted suspensions were treated with an ultrasonic bath (Model 3510, Branson, MS, USA) prior to the TEM operation. A droplet (5 μL) of diluted suspension was negatively stained with a droplet (5 μL) of 2 wt % uranyl acetate for about 2 min to enhance the contrast of the TEM images. The mixture was then immediately deposited on the surface of a 400-mesh carbon-coated copper grid. The excess liquid on the grid was absorbed using a tiny piece of filter paper to touch the edge of the grid. The morphologies of the obtained CNPs-48 and CNPs-64 were characterized using a transmission electron microscope (JEOL 100CX, JEOL, Inc., Peabody, MA, USA) with an accelerating voltage of 80 kV. The particle dimensions were calculated from the TEM images using ImageJ 1.45k software [23] according to a previously reported method [24]. For each sample, one hundred particles were randomly selected and measured from several TEM images.

### 2.6. Optical Transmittance Tests

The optical transmittances of PMMA and CNP/PMMA films were measured from 400 to 800 nm using a UV-vis spectrophotometer (Model EVO600PC, Thermo Fisher Scientific Inc., Pittsburgh, PA, USA). Transmission spectra were measured using air as a reference. The sample size was 50 mm ×12 mm × thickness. Three replicates were tested for each type of composite.

### 2.7. Wide-Angle X-ray Diffraction (WXRD) Tests

WXRD patterns of the samples were measured using a D/MAX 2200 X-ray diffractometer. The WXRD data were generated by a diffractometer with Cu Kα radiation (λ = 1.542 Å) at 40 kV and 30 mA over the angular range 2θ = 5°–40°, a step size of 4°/min. The degree of crystallinity or crystallinity index (*CI*, %) for each sample was evaluated using Equation (1) [25]:

$$CI=(A_{c}/A_{a})\times 100\text{\%}$$ (1)

where *A*_c_ is the area of the crystalline reflection and *A*_a_ is the area subtending the whole diffraction profile.

### 2.8. Differential Scanning Calorimetry (DSC) Tests

DSC runs using a TA Instrument Q200 differential scanning calorimeter (TA Instrument Inc., New Castle, DE, USA) were carried out to investigate the effect of CNPs on the glass transition temperature of the manufactured materials. Each scan was carried out under the nitrogen atmosphere (50 mL/min). To eliminate the thermal history, each sample (about 10 mg) was firstly heated from room temperature to 160 °C, which was followed by cooling down to 0 °C with 40 °C/min cooling rate. After maintaining at the isothermal condition for 5 min, the sample was heated to 160 °C at 10 °C/min. The glass transition temperature (*T*_g_) was defined as the mid-point of the change in the heat capacity on the heat flow and temperature plots of the second scan.

### 2.9. Coefficient of Thermal Expansion (CTE) Tests

Thermal expansion measurements were performed on a TMA Q400 thermomechanical analyzer (TA Instrument Inc., New Castle, DE, USA) in the tensile mode. Specimens were 25 mm long and 3 mm wide with a 20-mm effective length between two grips. Measurements were conducted under a nitrogen gas flow from 0 to 30 °C with a heating rate of 0.5 °C/min. The CTE was evaluated in the temperature range from 10 to 30 °C. Five replicates were tested for each condition.

### 2.10. Mechanical Property Measurements

Dynamic mechanical analysis (DMA) of PMMA and CNP/PMMA composites was carried out with a dual-cantilever mode using a TA Q800 analyzer (TA Instrument Inc., New Castle, DE, USA). The measurements were performed from room temperature to 140 °C at a constant frequency of 1 Hz, a strain amplitude of 0.01%, and a heating rate of 2 °C/min. Three replicates with dimensions of 60 mm in length and 12 mm in width were tested for each type of composite. The storage moduli of CNP/PMMA composites at different CNPs loadings were analyzed

The tensile properties of PMMA and CNP/PMMA composites were measured using a universal mechanical testing machine (CMT 4204, SANS Inc., Shenzhen, China). The load cell capacity was 100 N. Each specimen was cut into a length of 50 mm and a width of 3 mm. The gauge length and loading rate were controlled at 30 mm and 1 mm/min, respectively. The reported tensile modulus and strength values were averages from five samples.

## 3. Results and Discussion

### 3.1. Morphology of CNPs

**Figure 1.**
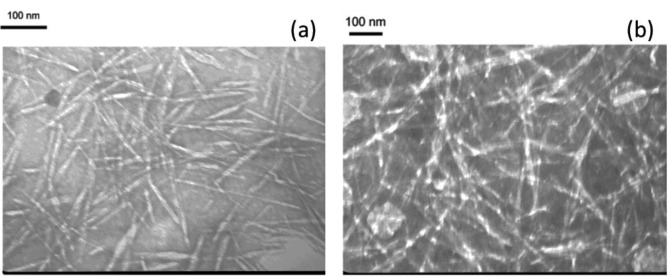
TEM observation of manufactured cellulose nanoparticles (**a**) (CNPs)-64 and (**b**) CNPs-48 material.

Figure 1a reveals that homogenized CNPs after treatment with 64 wt % H_2_SO_4_ followed by a HPH process were well-isolated and exhibited a rod-like structure (wider in the middle than at the ends), which is of a typical observation of well-dispersed CNPs in water. The average length and width of homogenized CNPs-64 were 152 ± 30 and 10 ± 3 nm, respectively. These values are consistent with those of previous reports [21,26,27] on cellulose nanocrystals. The individual nature of CNPs-48 after being subjected to the combined treatment of 48 wt% H_2_SO_4_ and homogenization was clearly observed in Figure 1b, where the CNPs-48 were oriented longitudinally in bundles. The average length and width of CNPs-48 were estimated to be 720 ± 210 and 19 ± 9 nm, respectively. Compared with the previously reported size of cellulose nanofibers produced from wood [28], the width of the obtained CNPs was similar, but their length was slightly shorter. The corresponding aspect ratios of CNPs-64 and CNPs-48 were 15.2 and 37.9, respectively.

### 3.2. Composite Appearance and Optical Transmittance

**Figure 2.**
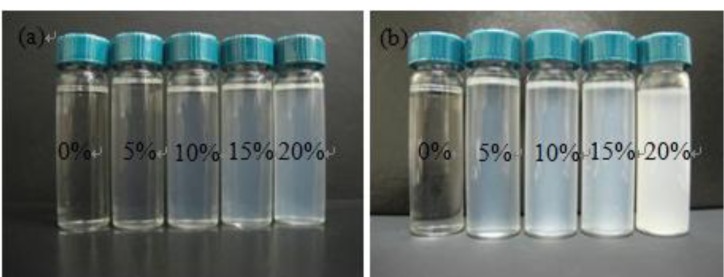
Polymethylmethacrylate (PMMA) and CNP/PMMA suspensions in dimethylformamide (DMF) at different loadings of (**a**) CNPs-64 and (**b**) CNPs-48.

**Figure 3.**
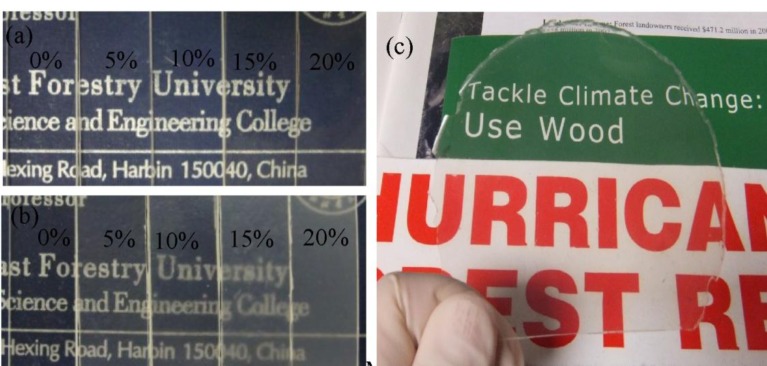
Photographs of pure PMMA and CNP/PMMA composite films placed on a background paper (**a**: CNPs-64, **b**: CNPs-48), and the CNP/PMMA composite film with (**c**) 20 wt% CNPs-64.

Photographs of CNP/PMMA suspensions in DMF solvent and CNP/PMMA composite films placed on a background paper are presented in Figures 2 and 3, respectively. As shown in Figure 2, PMMA formed clear solution in DMF. With the addition of CNPs to the system at low loading levels (e.g., 5 wt% and 10 wt%), the suspension was still transparent, especially for CNPs-64/PMMA composite. Further increase of CNP loadings led to increasingly opalescent suspensions, which is particularly true for CNPs-48/PMMA system due to the larger particle size of CNPs-48 material.

At the 20 wt% CNP loading level, the appearance difference of the CNPs-64/PMMA and CNPs-48/PMMA suspensions was much more obvious. The pattern and letters in the background can be clearly seen through the manufactured films in Figure 3. Samples with increased CNP loadings became more opalescent. The CNP/PMMA nanocomposite with a loading level of CNPs-64 as high as 20 wt% still possessed moderate transparency.

**Figure 4.**
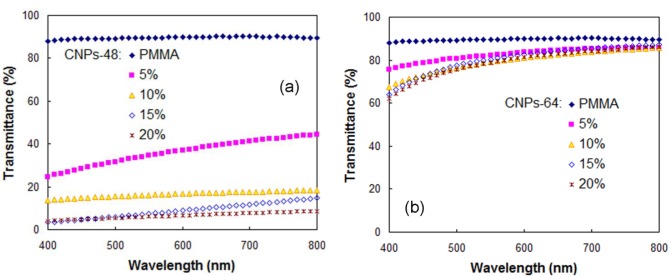
UV-vis transmittance spectra of pure PMMA and CNP/PMMA composites reinforced with (**a**) CNPs-48 and (**b**) CNPs-64.

The UV-vis transmittance spectra of pure PMMA and CNP/PMMA composite films at a visible wavelength range of 400 to 800 nm are shown in Figure 4. The CNPs loading had an influence on the optical transmittance for the CNP/PMMA nanocomposites, especially for CNPs-48/PMMA composite. The optical transmittance was reduced with increasing CNP loading for both CNPs-64/PMMA and CNPs-48/PMMA composites, probably due to the agglomeration of CNPs, especially at higher loading levels. CNPs-64/PMMA showed a significantly higher optical transmittance compared with the corresponding value for CNPs-48/PMMA at the same CNP loading. This phenomenon indicated that the optical properties of the CNP/PMMA nanocomposites were largely dependent on the difference in size and dispersion state of CNPs in the PMMA matrix.

### 3.3. WXRD Data

**Figure 5.**
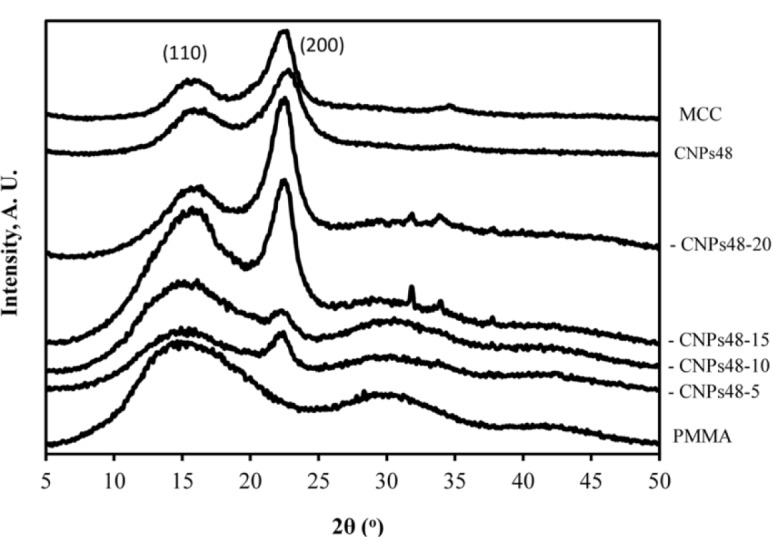
X-ray diffraction patterns of pure PMMA, pure cellulose materials and CNPs-48/PMMA composites at different CNP loading level.

The WXRD profiles of pure PMMA (DMF-processed), pure cellulose material, and CNPs-48/PMMA composites are presented in Figure 5. The neat PMMA exhibited two obvious diffraction peaks at 15.3° and 30.1°. The PMMA material used was mostly amorphous as there was no crystalline peak (Figure 5-PMMA curve). For a mixture of PMMA and CNPs, each component presents its own diffraction peaks in the composite. With the addition of crystalline CNPs to the amorphous PMMA matrix, the observed diffraction peaks of PMMA became weaker and crystalline peaks gradually appeared. This phenomenon could be explained by the fact that CNPs in the composites interacted with the hydroxide radical of the PMMA, leading to a good compatibility and strong interactions between them. There were very weak crystalline peaks for CNPs-48-5 and CNPs-48-10 composites, indicating the amorphous nature of these materials. However, the crystalline peak at 2θ = 22.5° became intensified when more CNP materials were added into the composites. The *CI* values of PMMA-CNPs-48-15 and PMMA-CNPs-48-20 increased to 20.8% and 26.8%, respectively. The *CI* value of CNPs-48 was 47.8%. With the increasing CNP loading level, the *CI* values of the composites increased, due to the high-crystalline structure of pure cellulose materials. Similar behavior was observed for the CNPs-64 and PMMA composites. The increased *CI* values for CNP/PMMA composites contributed to the strength of the composites as discussed later in the paper.

### 3.4. Thermal Expansion Properties

**Figure 6.**
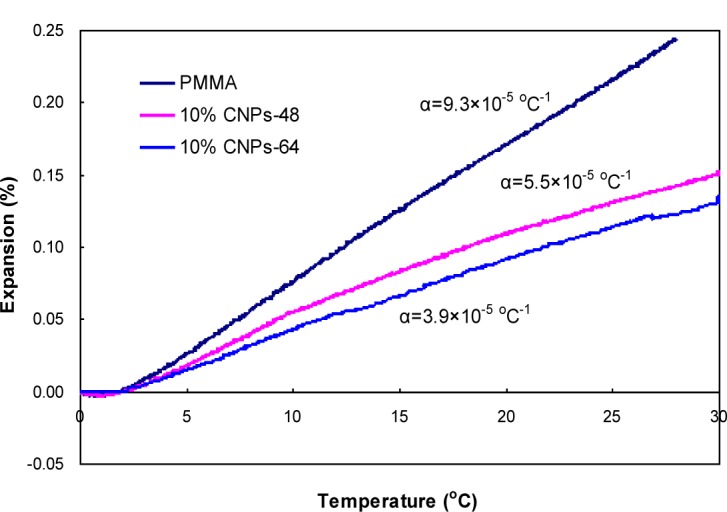
Comparison of thermal expansion between pure PMMA and CNP/PMMA composites.

CTE is one of the critical parameters in the selection of materials when designing thermal stable composite materials, such as electronic packaging. Figure 6 shows a comparison of the CTEs between pure DMF-processed PMMA and CNP-reinforced PMMA composites. The addition of 10 wt % CNPs to the PMMA matrix reduced the CTE from 9.3 × 10^−5^ to 5.5 × 10^−5^ and 3.9 × 10^−5^ °C^−1^ for CNPs-48 and CNPs-64, respectively. This decrease can be attributed to the low CTE of CNPs themselves. In addition, the thermal expansion reduction can also be attributed to filler/matrix interactions, as a strong interaction restricts the mobility of the polymer chains adhered to the filler surface [29]. CNPs-64/PMMA composites showed a slightly lower CTE than that of CNPs-48/PMMA. This result was probably due to the reduced size of the reinforcing elements. As reported by Vo *et al.* [30], the CTE of composites decreased with filler size. The addition of 10wt% CNPs-64 deteriorated the light transmittance by only 10% at a wavelength of 600 nm (Figure 4), a nearly 60% reduction of CTE (Figure 6).

### 3.5. Glass Transition Temperature

**Figure 7.**
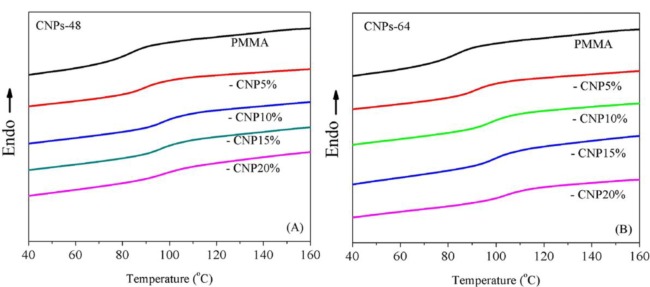
Effect of CNPs on the glass transition behavior of the composites. (**A**) CNPs-48/PMMA composites (*T*_g_ = 89.8, 96.8, 96.0, and 97.2 °C for 5, 10, 15 and 20 wt% CNPs); (**B**) CNPs-64/PMMA composites (*T*_g_ = 91.4, 97.4, 99.4, and 103.8 °C for 5, 10, 15 and 20 wt% CNPs).

Figure 7 illustrates the effect of CNPs on the glass transition temperature, *T*_g_, of the composites measured by DSC. It can be seen that incorporating CNPs played a positive role in modifying the glass transition temperature of DMF-processed PMMA (*T*_g-PMMA_ = 83.6 °C). The *T*_g_ of the composites increased with increased CNP loadings. This result was expected since CNPs could interact with the polymeric matrix and therefore hinder the rotation of polymeric chains, leading to the increased *T*_g_ [31].

In addition, it was interesting to notice that CNPs-64 exhibited a larger effect on modifying the *T*_g_ of the composites. For instance, the enhancement at the 15 wt% CNP level from the pure PMMA *T*_g_ value was 12.4 °C and 15.8 °C for CNPs-48 and CNPs-64 composites, respectively. This difference corresponded well with the mechanical properties discussed in the next section. It should be noted that when the CNP loading increased from 10 wt% to 20 wt%, a continuous increase in *T*_g_ was observed in CNPs-64 composites (from 97.4 °C to 103.8 °C). In CNPs-48 composites, however, the trend tended to level off (from 96.8 °C to 97.2 °C) for the same CNP loading level. This difference might be due to the different CNP structures (e.g., length and aspect ratio) between the two materials.

### 3.6. Mechanical Properties

**Figure 8.**
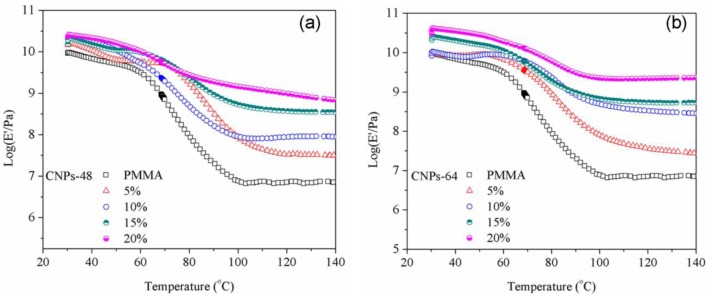
Temperature dependence of storage modulus (*E*′) for PMMA and CNP/PMMA composites reinforced with (**a**) CNPs-48 and (**b**) CNPs-64.

The storage moduli (*E*′) of CNP/PMMA composites at different CNP loadings are shown in Figure 8 and selected data are summarized in Table 1. It can be seen that the reinforcing effect of CNPs strongly depended upon the temperature and CNP level. At lower temperatures (Table 1 for 30 °C), the enhanced effect on storage modulus was somewhat limited at the low CNP loading levels (5 and 10 wt%) and became much more obvious at the higher CNP level (20 wt%). At higher temperatures (Table 1 for 100 °C), a monotonic increase in storage modulus was observed with increasing CNP loading level. The storage moduli of the composites reinforced with CNPs-64 at loading contents of 15 wt% and 20 wt% were about 518 MPa and 1341 MPa, respectively, compared with that of the DMF-processed PMMA (8 MPa at 100 °C). Such a difference could be attributed to the softened matrix at high temperatures [32].

At low temperatures, the PMMA matrix was very rigid, and therefore no drastic reinforcing effect from CNPs was observed especially at low CNP levels. While at higher temperatures (e.g., 100 °C which was above the *T*_g_ of the PMMA based on the DSC result), the matrix became softened. Thus, the reinforcement of CNPs was more obvious, since they could restrict the motion of the PMMA chains and therefore enhance the rigidity of the PMMA. In addition, CNPs-64 material seemed to be more effective in enhancing the modulus of the PMMA due to their better dispersion in the matrix, which is consistent with the results of static tensile modulus measurements.

**Figure 9.**
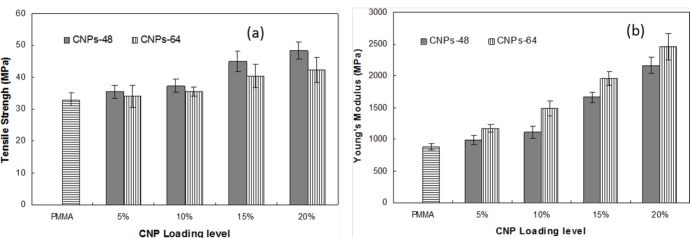
(**a**) Tensile strength and (**b**) Young’s modulus of pure PMMA and CNP/PMMA films at different CNP loadings.

Figure 9 shows the static tensile properties of pure DMF-processed PMMA and CNP/PMMA films at different CNP loading levels. All CNP/PMMA composites showed higher Young’s modulus and tensile strengths than those of the pure PMMA. The tensile properties were enhanced with increased CNP content in the PMMA matrix for both CNPs-64/PMMA and CNPs-48/PMMA. The composites with 20% CNPs possessed the highest values of tensile strength. The tensile strength of the composites reinforced with CNPs-48 at the 20 wt% loading level was 48.46 MPa, which was enhanced by 47% compared to that of the pure PMMA (32.99 MPa), indicating the reinforcement effect achieved from CNPs and the strong interaction between PMMA and CNPs. The CNPs-48/PMMA had higher tensile strength, but lower Young’s modulus than the CNPs-64-reinforced composites. The Young’s modulus of CNP/PMMA composites with 20 wt% CNPs-64 loading was 2455 MPa, which was increased by 180% compared to that of pure PMMA (880 MPa). Obviously, CNPs contributed more to the enhancement of the Young’s modulus than they did to the tensile strength. The significant improvement in tensile properties was probably due to the high strength (2 GPa) and elastic modulus (138 GPa) of the crystalline regions of cellulose I [5]. A previous study reported that the tensile strength and Young’s modulus of pure PMMA films were 27.6 GPa and 816 MPa, respectively, and that PMMA-based nanocomposites had a tensile strength of 44.1 MPa and a Young’s modulus of 2347 MPa [33]. The tensile strength and Young’s modulus of the pure PMMA film and the CNPs-64/PMMA composite at a loading level of 20 wt% are thus comparable with these previously reported data.

It was also observed that CNPs-48/PMMA composites were less brittle in handling and testing compared with CNPs-64/PMMA composites. This was attributed to the long fiber dimensions in the CNPs-48 material, which helped form a fiber network structure in the PMMA matrix. The differences in mechanical properties of the CNPs-64/PMMA and CNPs-48/PMMA composites could be related to the different characteristics of the two kinds of nanoparticles as CNPs-48 had higher aspect ratios than CNPs-64.

## 4. Summary and Conclusions

The transparent CNP-reinforced PMMA nanocomposites with high mechanical properties and low thermal expansion were successfully fabricated in this work. The addition of CNPs in the PMMA matrix decreased the optical transparency of the nanocomposites. The loss of light transmittance was limited to only 4.9% at 20 wt % CNPs-64 content at 600 nm wavelength. CNPs-48 resulted in a larger decrease in the optical transmittance compared to the CNPs-64 material. The coefficient of thermal expansion was significantly reduced by the addition of CNPs. CNPs-64/PMMA showed a slightly lower CTE than CNPs-48/PMMA. A strong reinforcement effect at higher temperature ranges was observed with the addition of CNPs into the PMMA matrix. The composite reinforced with CNPs-64 showed higher storage moduli compared to those with CNPs-48, especially at higher CNP loading levels. All CNP/PMMA composites showed higher static Young’s modulus and tensile strength than pure PMMA. The tensile properties were enhanced with increasing CNP content in the PMMA matrix. CNPs contributed more in improving the Young’s modulus than tensile strength.

## Figures and Tables

**Table 1.** Storage modulus (mean ± standard deviation) of CNP/PMMA composites at different CNP loadings.

CNP loading level	CNPs-64 (MPa)		CNPs-48 (MPa)	

	30 °C	100 °C	30 °C	100 °C
0	9204 ± 112	8 ± 1	9204 ± 112	8 ± 1
5	8994 ± 145	81 ± 11	14722 ± 148	87 ± 12
10	8269 ± 135	499 ± 23	21105 ± 167	91 ± 14
15	20635 ± 123	683 ± 27	15523 ± 193	518 ± 19
20	30324 ± 175	2267 ± 89	23357 ± 134	1431 ± 101
